# The Environment Makes a Difference: The Impact of Explicit and Implicit Attitudes as Precursors in Different Food Choice Tasks

**DOI:** 10.3389/fpsyg.2016.01301

**Published:** 2016-08-29

**Authors:** Laura M. König, Helge Giese, Harald T. Schupp, Britta Renner

**Affiliations:** Department of Psychology, University of KonstanzKonstanz, Germany

**Keywords:** explicit attitudes, implicit attitudes, food choice, choice architecture, dual process models

## Abstract

Studies show that implicit and explicit attitudes influence food choice. However, precursors of food choice often are investigated using tasks offering a very limited number of options despite the comparably complex environment surrounding real life food choice. In the present study, we investigated how the assortment impacts the relationship between implicit and explicit attitudes and food choice (confectionery and fruit), assuming that a more complex choice architecture is more taxing on cognitive resources. Specifically, a binary and a multiple option choice task based on the same stimulus set (fake food items) were presented to ninety-seven participants. Path modeling revealed that both explicit and implicit attitudes were associated with relative food choice (confectionery vs. fruit) in both tasks. In the binary option choice task, both explicit and implicit attitudes were significant precursors of food choice, with explicit attitudes having a greater impact. Conversely, in the multiple option choice task, the additive impact of explicit and implicit attitudes was qualified by an interaction indicating that, even if explicit and implicit attitudes toward confectionery were inconsistent, more confectionery was chosen than fruit if either was positive. This compensatory ‘one is sufficient’-effect indicates that the structure of the choice environment modulates the relationship between attitudes and choice. The study highlights that environmental constraints, such as the number of choice options, are an important boundary condition that need to be included when investigating the relationship between psychological precursors and behavior.

## Introduction

Coke and potato sticks is probably not a common breakfast food choice, but this eat “like a 6-year-old” diet has received a lot of public attention since Warren Buffett, an 84-year-old, top world investor, stated that this is his preferred breakfast choice^1^. Food choices and eating behavior show great diversity across people, and, with people making more than 200 food decisions a day, they represent a core aspect of our everyday life (Wansink and Sobal, 2007; see also Hofmann et al., 2012). Hence, actual eating behavior is likely to be driven by an interplay of explicit, or reflective, precursors (e.g., food attitudes, risk perceptions, self-efficacy, and behavioral intentions) and implicit, or automatic, reactions to stimuli in the environment which occur without conscious reflection (cf. Marteau et al., 2012).

That explicit and implicit attitudes play a significant role in explaining human behavior such as food choice is commonly accepted; this is reflected in a number of dual-process theories (e.g., Epstein, 1991; Sloman, 1996; Strack and Deutsch, 2004 and see Shiv and Fedorikhin, 2002; Ackermann and Mathieu, 2015 for consumer choices) and supported by results from various meta-analyses (e.g., Greenwald et al., 2003; Hofmann et al., 2005; Rooke et al., 2008; Reich et al., 2010). However, the question of how implicit and explicit processes work in conjunction to control an overt behavioral response is still under research. In their reflective-impulsive model (RIM), Strack and Deutsch (2004) suggest that these processes operate in parallel and compete for control of an overt behavioral response. Since the impulsive system requires little cognitive capacity, it is commonly assumed that its impact on behavior increases as conditions become more resource demanding (see also MODE Model; Fazio and Olson, 2003). Along these lines, it has been repeatedly shown that restrained cognitive resources (e.g., due to cognitive load, ego depletion) facilitate the influence of implicit attitudes on food choice (e.g., Shiv and Fedorikhin, 1999, 2002; Cervellon et al., 2007; Hofmann et al., 2007; Friese et al., 2008; see also Hofmann et al., 2009b; Sheeran et al., 2013). For example, implicit attitudes were more strongly related to the food choice observed (number of chocolates chosen) when participants were asked to remember an eight-digit number (high cognitive load) instead of a one-digit number (low cognitive load) (Friese et al., 2008; see also Shiv and Fedorikhin, 1999, 2002; Cervellon et al., 2007). Similarly, implicit attitudes were more strongly related to chocolate consumption when participants were asked to suppress their emotion while watching a video clip (ego depletion condition) as compared to when no emotion regulation instruction was given (control condition) (Hofmann et al., 2007).

However, past research has predominantly focused on boundary conditions related to either the person (e.g., habitualness, Conner et al., 2007) or additional behavioral demands (e.g., cognitive load, ego-depletion, Hofmann et al., 2009a), ignoring situational conditions such as the structure of the target behavior. Most studies assessing the impact of implicit and explicit attitudes on food choices realized a choice task where participants were asked to choose a specific food item. Richetin et al. (2007), for instance, offered participants a selection of fruits and snacks and asked them to choose one free snack or a piece of fruit as compensation for their participation (see also Perugini, 2005; Conner et al., 2007; Ayres et al., 2012; Holland et al., 2012). Using a similar behavioral choice set, Friese et al. (2008) asked the participants to select five items from a selection of fruits and chocolates. Thus, the structure of the target behavior was typically unvaried and its impact has yet to be assessed systematically (see also Perugini, 2005).

The structure of the target behavior, that is which types of behavioral choices are possible, is dependent on the food availability or assortment size (number of food options), which represents a core part of the ‘choice architecture’ (Thaler and Sunstein, 2008; see also Marteau et al., 2011) or ‘obesogenic food environment’ (Swinburn et al., 1999). We propose that the context of the behavioral choices, in particular the assortment size (number of food options), has a significant impact on the relative weight of implicit and explicit attitudes for food choices. In the real world, the context of food choices is rather complex. Today, German supermarkets typically offer more than 25,000 choices (EHI Retail Institute, 2014). Moreover, several decisions have to be made in parallel when grocery shopping or selecting dishes for a multiple course meal as not only the items but also the quantity has to be considered (Wansink et al., 2009). Considering that the complexity of behavioral options impacts the quality of and satisfaction with behavioral choices (Lee and Lee, 2004; Scheibehenne et al., 2010), it appears plausible to assume that the context of the target behavior is an important boundary condition that can shift the weight between implicit and explicit attitudes as precursors.

Building on previous work by Conner et al. (2007) investigating personal moderators of the relationship between explicit and implicit attitudes and snack choice, the present study aimed at examining the impact of implicit and explicit attitudes in a binary and a multiple option food choice context to broaden the knowledge about situational moderators. Specifically, assuming that resource demanding conditions increase the impact of implicit attitudes, we hypothesized that implicit measures of attitudes are better predictors of food choices in a multiple compared to a binary option choice task.

**FIGURE 1:**
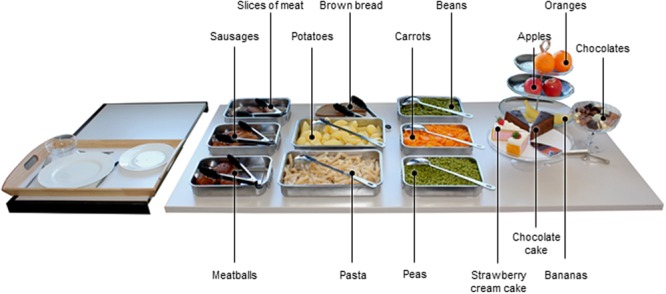
**Two course fake food buffet including 15 different food items for lunch**.

However, realizing a standardized target stimuli set for spontaneous food choices is challenging since it often conflicts with practical issues, for example, high costs, high preparation effort and waste. To overcome these problems, we applied a method recently developed by Bucher et al. (2012) using replica food items (**Figure 1**) (see also Sproesser et al., 2015). The Fake Food method has been shown to be reliable and a valid assessment of real food choices (Bucher et al., 2012). Using fake food, a multiple option context, such as eating in buffet style restaurants, can be simulated under well-controlled conditions. Moreover, the same food items can be used in multiple and binary options contexts, reducing variability due to different target stimuli sets.

## Materials and Methods

### Participants

A power analysis using G^∗^Power 3.1 (Faul et al., 2009) to detect a medium effect (*f*^2^ = 0.15), assuming three predictors in a linear multiple regression, yielded an *N* of 77 for 80% power. Ninety-seven participants (75% female) were recruited by e-mail using student mailing lists from the University of Konstanz, short notices distributed around the university and postings on Facebook groups. Potential participants were informed that omnivores (i.e., non-vegans and non-vegetarians) and people without health related dietary restrictions were eligible for the study. In addition, participants were asked on-site whether they were vegetarian, vegan or had any health related dietary restrictions. As none of the participants reported these limitations, all participants were included in the analyses. Participants were aged 19 to 46 (*M* = 23.22, *SD* = 4.49) and 97.9% were students, representing a wide range of subjects. As compensation, participants received either 12€ or 1.5 h of course credit.

### Procedure

The participants were invited to the laboratory for individual sessions. Prior to participation, all participants gave written informed consent in accordance with the Declaration of Helsinki. They were seated in front of a 19 inch computer screen and were asked to fill in a questionnaire assessing explicit attitudes. Afterward, a single category implicit attitude test (SC-IAT) was conducted followed by the binary food choice task. Participants were then asked to step in front of the fake food buffet that was covered by a table cloth. The experimenter asked the participants to serve themselves a meal that they would typically have for lunch, provided them with tableware and removed the table cloth. Participants were free to choose the contents and size of their meal (multiple food choice task). When the participants were finished, they were asked to place the dishes on the serving tray. In a final step, they filled in a second computer-based questionnaire assessing demographic and anthropomorphic variables, as well as eating motives that will not be discussed in the present publication. Finally, participants were thanked and paid. During the study, participants wore SMI eye tracking glasses to monitor their eye movements; however, eye movements and selective attention are not the focus of the current paper and are reported only in the interest of full disclosure.

### Materials

#### Food Stimuli: Fake Food Items

In total, 15 different food items from five food categories (vegetables, meat, side dishes, confectionery, and fruit) were used in the study (**Figure 1**). The same 15 stimuli were used both as realistic food replicas on a buffet and as pictures of the replicas in the questionnaire and the two computerized tasks (SC-IAT and binary option choice task). The food pictures had a size of 400 × 300 pixels and depicted one standard serving of the respective food item according to German dietary guidelines (Koelsch and Brüggemann, 2012).

All food items were pre-tested to ensure that each of the replica food items in the pictures were clearly identifiable and realistic. Twenty-two participants (68% female; age: *M* = 24.86, *SD* = 3.64) rated a total of 27 pictures of food replicas from the five food categories. Participants were asked to indicate what food item they saw in the picture and indicate to what extent the food item was identifiable and realistic on a four point Likert scale, with higher values representing a more positive evaluation. For each food category, three items that had been correctly identified by all participants and rated the most identifiable and realistic were selected (Supplementary Table 1). In order to increase comparability with previous studies on food choice, we focused on confectioneries and fruit in the main analyses, specifically an assortment of chocolates, chocolate or strawberry cream cake, and apples, bananas and oranges (**Figure 1**; Supplementary Table 1).

### Measures

#### Explicit Attitudes

Explicit attitudes were assessed using a computerized questionnaire. For each food item used in the study, a picture was presented at the top of the page followed by questions concerning the depicted food item. Participants were asked to indicate whether the depicted food item was tasty vs. not tasty, good vs. bad and appealing vs. unappealing on a six point semantic differential, with higher values representing a more positive evaluation. In addition, participants evaluated the healthiness (unhealthy vs. healthy) of the foods, but this item was not included in the scale because it reduced internal consistency (confectioneries: α = 0.83 with healthiness item versus α = 0.91 without healthiness item; fruit: α = 0.85 with healthiness item versus α = 0.93 without healthiness item). This concurs with previous research, which consistently finds a difference between palatability and health attitudes (Verplanken et al., 1998; Ayres et al., 2012). The three respective items, chocolates, chocolate, or strawberry cream cake as well as apple, banana, and orange, were aggregated to yield attitude scores for confectionery and fruit, respectively. The final attitude score was taken as the difference between the attitude toward confectionery and the attitude toward fruit (i.e., positive scores indicate a preference for confectionery over fruit).

#### Implicit Attitudes

A SC-IAT was implemented in Presentation software (Version 17.0^2^) and conducted following the procedure described by Karpinski and Steinman (2006). The stimulus material consisted of 15 fake food pictures and six positive and six negative pictures taken from the IAPS database (Lang et al., 1999)^3^ (see also Hofmann and Friese, 2008 for a similar procedure). The IAPS pictures were selected on the basis of the ratings provided by Lang et al. (1999). The positive and negative picture sets were comparable regarding arousal (*M*_Positive_ = 5.46, *SD*_Positive_ = 0.53; *M*_Negative_ = 5.6, *SD*_Negative_ = 0.92). They differed significantly regarding valence ratings (*M*_Positive_ = 7.7, *SD*_Positive_ = 0.50; *M*_Negative_ = 2.43, *SD*_Negative_ = 0.48; *t*(10) = 18.61, *p* < 0.001), but the valence ratings were equally extreme, as shown by the absolute z-standardized values (*M*_Positive_ = 1.5, *SD*_Positive_ = 0.28; *M*_Negative_ = 1.47, *SD*_Negative_ = 0.27).

The SC-IAT consisted of eleven blocks in total. For each block, participants were instructed to categorize the images as accurately as possible by pressing one of two keys. The category labels were presented as reminders at the top of the screen during the task. Whenever participants made an error in categorizing, they were presented with a 50 ms black screen before they were informed about the error by a red cross and asked to correct the error by pressing the other key. Reaction times were recorded. If no reaction occurred within 10 s after trial onset, the trial was aborted and the next trial started. Between trials, there was an intertrial interval of 300 ms. Raw data was scored according to the recommendations of Greenwald et al. (2003), including the calculation of *D*-scores with a positive score representing a stronger association with the positive category.

The first block consisted of 24 trials and served as a training phase where the participants were familiarized with the positive and negative IAPS pictures to ensure that they were able to correctly categorize images as ‘positive’ or ‘negative.’ Afterward, the food pictures and categories were added in the following 10 blocks, with two blocks per food category. The present publication focuses on the four blocks for the categories confectionery and fruit. The two target categories were coupled with the category ‘negative’ in the first block and ‘positive’ in the second block. Each block consisted of 42 trials with each picture appearing at least twice. Similar to the explicit attitudes measure, a final score for implicit attitudes was obtained by subtracting the *D-*score for fruit (α = 0.67) from the *D*-score for confectionery (α = 0.66), with a positive value reflecting a preference for confectionery over fruit.

#### Binary Option Choice Task

**FIGURE 2:**
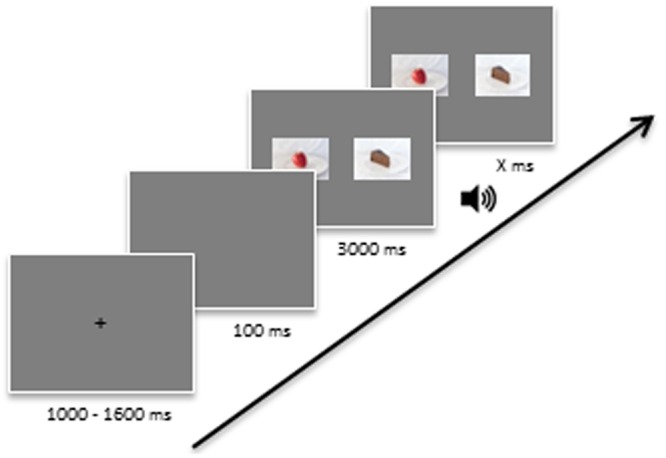
**Flow chart of the binary option choice task**.

In order to assess food choices in a binary option context, participants were presented with two fake food pictures per trial using Presentation software (Version 17.0^2^). The two presented food items were centered vertically on a gray background on the computer screen with a 6 cm distance between the inner edges of the two images. The presentation procedure followed Graham et al. (2011). First, a 1.000 to 1.600 ms jittered interval with a black fixation cross was displayed in the center of the screen, followed by a 100 ms blank screen. Then, the two food pictures were presented simultaneously. Participants watched freely for three seconds before being asked to choose which of the two food items they would prefer to eat by pressing a key (**Figure 2**).

In total, all 15 fake food items were compared with one another over 105 trials, leading to a choice score per item ranging from 0, where all other food items were preferred to this item, to 14, where no other food item was preferred to this item. To obtain sum scores for the categories, all comparisons between an item and items from each of the other categories were summed up, thus reducing the possible maximum number of choices per item to 12. The scores per item then were summed up to yield a score per category, leading to choice scores per category from 0 to 36. A food choice difference score was created by subtracting the score for fruit from the score for confectionery. Positive scores indicate that confectionery was chosen more often than fruit.

#### Multiple Option Choice Task

To create a realistic, multiple option context to investigate food choice, a fake food buffet was composed according to Bucher et al. (2013). In total, the lunch buffet included 15 different foods which were placed in serving bowls and arranged on a table to resemble an actual buffet (**Figure 1**). Participants were provided with a serving tray (55 cm × 35 cm) containing a large (27 cm diameter) and a small plate (21 cm diameter) as well as a small bowl (12 cm diameter) and were asked to serve themselves a meal which they would usually eat for lunch. Pictures showing examples for the meals self-served can be found in Supplementary Data Sheet 1. After the participants left the laboratory, the components of the meals were weighted (continuous items, e.g., pasta) or counted (e.g., chocolate candies) by the experimenter. Before analyzing the data, the weight of each food replica was converted into the respective weight of the real food by multiplying the amount of each replica with a predetermined factor based on a comparison of the replica item and the respective real item. The energy estimates and nutrient content of each self-served food item was calculated using the German food database Bundeslebensmittelschlüssel (Hartmann et al., 2005). To yield the total amount of self-served confectionery in grams, the values for the food replica items chocolate cake, chocolates, and strawberry cream cake were summed up. Similarly, the amount of fruit was computed by summing up the amount of self-served apples, bananas, and oranges. To obtain a food choice score, the converted amount of self-served fruit was subtracted from the converted amount of self-served confectionery (see also Conner et al., 2007 for a similar procedure). Thus, a positive choice score indicates that more confectionery than fruit was chosen in grams.

#### Body Mass Index

Body Mass Index (BMI) was calculated based on self-reported weight and height.

#### Hunger

Participants were asked to indicate their hunger on a six point Likert scale ranging from (1) very hungry to (6) very full.

### Statistical Analysis

To analyze the relationships between explicit and implicit attitudes and food choices in the binary and multiple option conditions, a path model analysis was conducted in Mplus 7.1 (Muthén and Muthén, 2012). For explicit attitudes, there were missing values for two participants (2.1%). To estimate missing values, full information maximum likelihood (FIML) estimates were applied (Muthén and Muthén, 2012) that estimate missing values based on the observed model variables. This method has been shown to be robust and more efficient than other missing value estimation methods (Enders and Bandalos, 2001).

## Results

Descriptive statistics as well as zero-order correlations between study variables are depicted in **Table 1**.

**Table 1:** Descriptive statistics and zero-order correlations between study variables.

		*M*	*SD*	2	3	4	5	6	7	8
1	Explicit attitudes	-0.67	1.15	0.04	0.63^∗∗^	0.31^∗∗^	-0.21^∗^	-0.06	0.13	0.03
2	Implicit attitudes	-0.20	0.42		0.22^∗^	0.29^∗∗^	-0.10	-0.21^∗^	0.07	-0.03
3	Binary option context	-1.55	4.28			0.50^∗∗^	-0.03	-0.11	0.09	-0.25^∗^
4	Multiple option context	107.00	216.83				-0.05	-0.19	0.08	-0.22^∗^
5	Age	23.22	4.49					0.27^∗^	-0.03	-0.22^∗^
6	BMI	22.21	2.87						0.03	0.15
7	Gender^1^	(25 %)								-0.23^∗^
8	Hunger	3.58	1.15							

*Multiple option context: weight of food replicas converted to weight of real food (in grams), reported as a difference score for confectionery versus fruit. Gender: 0 = female, 1 = male. ^1^For Gender, the proportion of males in the sample is reported instead of the mean. ^∗^*p* < 0.05, ^∗∗^*p* < 0.01.*

On average, participants held a more positive explicit attitude toward fruit than confectionery (*M* = -0.67, *SD* = 1.15). The SC-IAT also indicated a more positive implicit attitude toward fruit (*M* = -0.20, *SD* = 0.42). Explicit and implicit attitudes were not significantly correlated [*r*(95) = 0.04, *p* = 0.720].

In the binary option context, participants on average chose fruit more often than confectionery (*M* = -1.55, *SD* = 4.28). While confectionery was chosen in 7.41 (*SD* = 3.33) out of 36 comparisons, fruit was chosen in 8.96 (*SD* = 2.38) out of 36 comparisons. In the multiple option context, participants served themselves on average 153.86 (*SD* = 93.93) grams of fruit and 53.32 grams (*SD* = 56.05) of confectionery. On average, they selected 107.0 grams more confectionery than fruit (*SD* = 216.83). Of the total meal, confectionery accounted for 8.43% (*SD* = 8.51) of the total real weight (grams) and 21.98% (*SD* = 19.99) of the energy (kcal), while fruit accounted for 25.20% (*SD* = 14.32) of the total real weight and 13.81% (*SD* = 10.60) of the energy. Together, they accounted for 33.64% (*SD* = 14.7) of the total real weight and 35.79% (*SD* = 18.94) of the energy of the meals self-served.

### Food Choice

**FIGURE 3:**
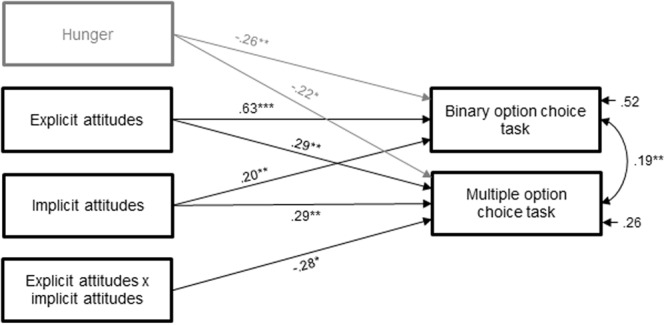
**Path diagram of the associations between explicit and implicit attitudes and confectionery choice in the two choice tasks option contexts**. ^∗^*p* < 0.05, ^∗∗^*p* < 0.01, ^∗∗∗^*p* < 0.001.

To test the relationship between implicit and explicit attitudes and food choice (confectionery versus fruit) in the two different choice tasks, a path analysis was performed (**Figure 3**). Hunger was included as a control variable.^4^

In the binary option choice task, the amount of confectionery versus fruit chosen significantly increased as a function of explicit attitudes (β = 0.63, *p* < 0.001, 95% CI [0.46; 0.77]). Also, the positive relation between implicit attitudes and confectionery choice reached statistical significance (β = 0.20, *p* = 0.003, 95% CI [0.07; 0.33]). The interaction between explicit and implicit attitudes was not significant (β = -0.11, *p* = 0.319, 95% CI [-0.33; 0.09]). Furthermore, hunger was negatively associated with the amount of confectionery versus fruit chosen (β = -0.26, *p* = 0.003, 95% CI [-0.44; -0.10]). Together these variables explained 51% of the variance in food choice in the binary option choice task.

**FIGURE 4:**
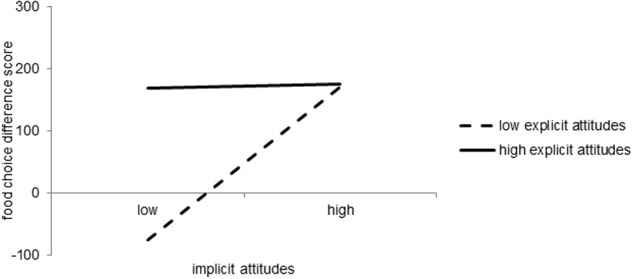
**Simple effects of attitudes on food choice in the multiple option choice task.** Simple slope effects for | 1 SD| for both explicit and implicit attitudes. Food choice is reported as the food choice difference score.

Turning to the multiple option choice task shows that the amount of confectionery versus fruit chosen from the buffet increased as a function of explicit (β = 0.29, *p* = 0.002, 95% CI [0.11; 0.49]) as well as implicit attitudes (β = 0.29, *p* = 0.001, 95% CI [0.14; 0.47]). The two main effects were qualified by a significant interaction between explicit and implicit attitudes on food choice (β = -0.28, *p* = 0.026, 95% CI [-0.52; -0.03]). As **Figure 4** depicts, a greater amount of confectionery was chosen on average when both attitudes showed a consistent preference for confectionery. However, a greater amount of confectionery was also chosen when explicit and implicit attitudes toward confectionery were inconsistent (i.e., one attitude type indicated a stronger preference for confectionery while the other indicated a stronger preference for fruit), indicating a compensatory ‘one is sufficient’-effect. Conversely, participants only chose more fruit on average when both attitude types consistently indicated a greater preference for fruit. Accordingly, simple effects revealed that food choice differed significantly between participants with low (-1 SD) and high (+1 SD) implicit attitudes (β = 0.29, *p* = 0.003) when explicit attitudes were 1 SD below the mean. However, food choice did not differ between participants with low or high implicit attitudes (β = 0.01, *p* = 0.925) when explicit attitudes were 1 SD above the mean. Moreover, hunger was negatively associated with the amount of confectionery versus fruit chosen (β = -0.22, *p* = 0.022, 95% CI [-0.41; -0.02]). Together, the included variables explained 26% of the variance in food choice in the multiple option context^5^.

To further test the relative impact of implicit and explicit attitudes across the two choice tasks, we additionally compared the path coefficients. Comparing the two attitude-food choice path coefficients within the respective choice task shows that the impact of implicit attitudes was significantly weaker than the impact of explicit attitudes in the binary option choice task [χ^2^ (1, *N* = 97) = 15.74, *p* < 0.001], while the impact of implicit attitudes was comparable to the impact of explicit attitudes on food choice in the multiple option choice task [χ^2^ (1, *N* = 97) = 0.00, *p* = 0.996]. Explicit attitudes were more strongly associated with confectionery choices in the binary option choice task [χ^2^ (1, *N* = 97) = 11.70, *p* < 0.001]. Regarding implicit attitudes, the relationships in both choice tasks did not differ significantly [χ^2^ (1, *N* = 97) = 0.94, *p* = 0.332].

## Discussion

In the present study, the relationships between explicit and implicit attitudes and food choice (confectionery vs. fruit) were investigated in two different choice tasks. In the binary option choice task, both types of attitudes were significant precursors of food choice. However, explicit attitudes clearly had a greater impact. Conversely, in the multiple option choice task, the additive impact of explicit and implicit attitudes was qualified by an interaction indicating that more confectionery was chosen even if explicit and implicit attitudes toward confectionaries were inconsistent and one of them was positive. Therefore, the results support the notion that the context of the target behavior is a significant boundary condition which shifts the balance between implicit and explicit attitudes as precursors within choice environments.

### Shift between Implicit and Explicit Attitudes as Precursors

Inherent in dual process models (e.g., Fazio, 1990; Strack and Deutsch, 2004) is the assumption that the degree of taxes on cognitive resources shifts the weight between a reflective and an impulsive system, which may lead to the activation of different behavioral schemata (e.g., Strack and Deutsch, 2004; Hofmann et al., 2008). In the literature (Perugini, 2005; see also Cervellon et al., 2007) three different models describing the interplay of explicit and implicit determinants are discussed: The additive model assumes that implicit and explicit attitudes independently predict the target behavior, the double dissociation model proposes that implicit attitudes predict spontaneous whereas explicit attitudes predict deliberative behavior and the multiplicative model assumes that implicit and explicit attitudes interact in predicting the target behavior. According to the dissociative pattern, explicit precursors shape behavior in some situations, while implicit precursors shape behavior in others (Perugini, 2005). As, in the present study, explicit and implicit attitudes co-varied with food choice in both choice option contexts, the results support the additive model, suggesting that both explicit and implicit attitudes independently contribute to snack choice and explain differing variance in the target behavior. This pattern of results is in line with previous studies showing that both explicit and implicit attitudes were related to sweet versus fruit choice (e.g., Conner et al., 2007). However, in the multiple option choice task, the main effects of implicit and explicit attitudes on food choice were qualified by an interaction between the attitude measures, also supporting the multiplicative pattern. As predicted, the results showed that participants served themselves a greater amount of confectionery compared to fruit when implicit and explicit attitudes indicated a preference for confectionery over fruit. Interestingly, more confectionery than fruit was also chosen when explicit and implicit attitudes toward confectionery were inconsistent and one was positive. Only when both attitudes indicated a preference for fruit over confectionery did participants serve themselves more fruit than confectionery. Hence, the overall pattern of results in the multiple option choice task seems to indicate a compensatory ‘one is sufficient’-effect.

While most studies that investigated the relationship between implicit and explicit attitudes and food choice did not include the interaction in their analysis, the studies that tested the multiplicative model have yet to report significant results (Perugini, 2005; Richetin et al., 2007; Spence and Townsend, 2007; Friese et al., 2008). However, the measures these studies applied to assessing food choice are more comparable to the binary option choice task in the present study, where the interaction also did not reach significance. For instance, Perugini (2005) and Richetin et al. (2007) assessed snack choice by offering participants two alternatives to choose from, while Spence and Townsend (2007) offered genetically modified cookies and assessed whether participants accepted them. The most similar measure was used by Friese et al. (2008, Study 2), where participants were allowed to select five items out of a box that contained a selection of two chocolate bars and two kinds of fruit. As the amount if items was fixed and the number of available choice alternatives was lower (four overall compared to six deserts in addition to nine main meal foods in the present study), we argue that the choice environment provided by Friese et al. (2008) is still more limited than the multiple option choice task realized in the present study. From the literature and the present study, a clear threshold between a binary and a multiple option setting causing the two observed patterns cannot be derived, thus it is open to future studies to examine this question in greater detail.

### The Environment Makes a Difference

To investigate shifting between implicit and explicit attitudes as precursors, one behavior is examined under different circumstances by taking dispositional or situational moderators into account. Thus, to investigate the relationship between explicit and implicit attitudes and food choice, one also needs to ask when they are associated in addition to whether they are associated in general (c.f. Perugini, 2005). Previous studies have focused on examining whether explicit and implicit attitudes are related to food choice in general, thus assessing food choice with only one measure, e.g., daily snacking recorded in a diary (Ayres et al., 2012, study 1; Conner et al., 2007, study 1) or choice of sweets versus fruits in a behavioral choice task (Richetin et al., 2007; Prestwich et al., 2011). The present study adds to this line of research by comparing two choice tasks that differ in structure and showing a shift from a strong explicit and weaker implicit precursor of food choice to two interacting precursors in a multiple and thus more complex choice environment. Similar to studies inducing cognitive load by an additional task, e.g., memory tasks (Friese et al., 2008, Study 1) or emotion suppression (Hofmann et al., 2007), one might argue that cognitive demands varied between the two choice tasks. In the binary option choice task, decisions were sequential and choices between trials unrelated while the multiple option choice task required numerous parallel decisions: whether to have or abstain from having a dessert, which combination of the six different available cakes, candies, and fruits to select and how much confectionery to choose. Although multiple comparisons were made in the binary option choice task and the task required more time to complete than the multiple option choice task, tiredness, or fatigue might be unlikely to have influenced the results in the present study as a recent analysis of respondent fatigue in choice experiments has reported inconclusive results across several data sets (Hess et al., 2012). Although fatigue induced by the choice tasks was not assessed in the present study, the observed pattern of results might support this notion: if cognitive resources had been depleted by the task, a stronger relationship between food choice and implicit attitudes would have been expected based on the literature (e.g., Friese et al., 2008, Study 1). Thus, it is likely that cognitive resources were more taxed in the multiple option choice task than the binary option choice task due to the need to make several decisions simultaneously (Scheibehenne et al., 2010). However, since the fake food buffet might have been more realistic than the binary and picture based choice task, the food items might have provided a greater challenge for self-control resources due to temptation.

From the choice tasks compared in the present study, a parallel can be drawn to food choice contexts in daily life that may also differ in cognitive and self-control demands. In situations where only limited information is available or the number of available foods to choose from is limited, more cognitive resources may be available for deliberate decision making. In a recent point-of-purchase intervention study, Allan et al. (2015) showed that decreasing cognitive demands during choice by providing easily accessible healthiness information decreased the purchase of high calorie snacks. On the other hand, in situations with a large number of choice alternatives, e.g., when selecting foods from a cafeteria buffet or restaurant menu, the increased number of alternatives may increase the cognitive load, leading to a possible shift in the weight between reflective and impulsive precursors and subsequent overeating due to reduced cognitive control (see also Swinburn et al., 1999, 2011; Hardman et al., 2015). This notion is supported by observational studies investigating the link between eating out and BMI, which suggest that in less controlled environments that potentially provide more opportunities to eat and a greater selection of foods like restaurants increase obesity (Ma et al., 2003; Bezerra et al., 2012). The same relationship has been observed for supermarkets: the more supermarkets are available within a neighborhood, the higher the residents’ BMI (Wang et al., 2007), and people who consistently shop at the same supermarket have been shown to have a more similar BMI (Chaix et al., 2012).

Environmental influences on eating behavior have recently gained a lot of attention. These studies suggest an impact of the eating environment on the evaluation of food items or foods chosen (Hollands et al., 2013; for reviews, see Wansink, 2004; Cohen and Babey, 2012). For example, changes in the physical environment, e.g., in the accessibility of foods (Wansink et al., 2006; Rozin et al., 2011; Kroese et al., 2015) facilitate healthier food choices. These studies, however, are silent regarding the processes underlying the different choices and thus have not investigated the moderating role of the environment on the relationship between precursor and behavior. The present study adds to this line of research by showing that changes in the choice architecture also modulate the relationship between precursor and behavior. The results highlight the need for a more systematic investigation of the relationship between a precursor and a behavior in different contexts. It can thus be suggested that health behavior interventions targeting motivation or other socio-cognitive variables might also be context-dependent. Therefore, they underline the need to tailor interventions not only to the characteristics of the target population (e.g., Schwarzer, 2008) but also to the characteristics of the context in which they are applied.

### Limitations

The present study used two tasks to assess food choice. The two tasks differed in sensory stimulation and the number of options presented simultaneously. Whereas 2D pictures were displayed on a computer screen in the binary option choice task, the multiple option choice task presented 3D objects that had to be touched by the participants. The multiple option choice task thus increased cognitive load through the combination of a greater stimulus array. To disentangle the effects of choice assortment and sensory stimulation, fakes foods could be used in both choice tasks in future studies. However, realizing a sufficient number of trials for the binary option choice task might be challenging since an experimental set up needs to be realized where choice tasks are presented in a timely manner without participants glimpsing previous or upcoming tasks. Another limitation might be that the weight of food replica and real food diverges. However, a study which invited participants on two separate occasions showed a high agreement between self-served meals from a real food buffet and a fake food buffet (*r* = 0.77 to *r* = 0.93; Bucher et al., 2012) suggesting a high validity of the fake food buffet method.

Regarding the study design, further limitations need to be acknowledged. The two choice tasks differed slightly in the instructions given. While participants were asked to choose which food they prefer in the binary option choice task, they were asked to choose what they typically eat for lunch in the multiple option choice task. Although the differences in instructions were subtler in the present study than in previous research examining task instruction differences on reaction times and eye movements during food choice (e.g., van der Laan et al., 2015), we cannot preclude the possibility that the differences influenced the relationship between explicit attitudes and food choice. Furthermore, the two choice tasks were not counterbalanced, thus choices in the binary option choice task may have influenced choice in the multiple option choice task due to consistency strivings. Furthermore, the present study was conducted in a laboratory setting, raising the question whether the found effects generalize to food choice in daily life. Two previous studies investigating the relationship between explicit and implicit attitudes and food choice in real life found associations between snack choice and explicit attitudes but not implicit attitudes (Conner et al., 2007; Ayres et al., 2012). This might indicate that, in real life, explicit attitudes are more strongly related to snacking than implicit attitudes. Alternatively, one might argue that the different pattern of results is due to the different methodological approaches used to assess the target behavior. For example, Ayres et al. (2012) used a relative consumption score (daily ratio of chocolate to fruit, with scores over 0.5 indicating that more than half the choices were for chocolate, see also Conner et al., 2007, Studies 1 and 2; Perugini, 2005, Study 2 for a similar approach). Averaged, relative consumption scores over many different types of eating occasions and environments might conceal the relationship between the variables. While situational characteristics can be controlled and manipulated in the laboratory, diary studies have yet to assess situational characteristics like choice assortment. To overcome this problem, future studies could use Ecological Momentary Assessment (Shiffman et al., 2008; Schüz et al., 2015), allowing a food diary to be combined with context measures.

The sample of the present study mainly consisted of female undergraduate students without any dietary restrictions, thus it has to be acknowledge that the generalizability of the findings is limited. Although female students are a common population in previous studies on the relationship between reflective and impulsive influences and food choice (e.g., Perugini, 2005; Conner et al., 2007; Richetin et al., 2007; Friese et al., 2008; Ayres et al., 2012) and the use of the same study population enhances comparability of the findings, it would be desirable to replicate the findings with more diverse samples to enhance generalizability.

## Conclusion

The results of the present study demonstrate that different food choice tasks might lead to different conclusions about how food choices are made. Thus, choice environments and their ‘choice architecture’ offering different kinds of choice assortments might only be comparable to a limited extent. Both explicit and implicit attitudes were related to food choice in both tasks, with explicit attitudes being more strongly related to food choice in the ‘less demanding’ binary option choice task than implicit attitudes. Importantly, in the ‘more complex,’ multiple option choice task, an interaction between implicit and explicit attitudes emerged, indicating a compensatory ‘one-is-sufficient’ effect that triggers an ‘unhealthy’ choice pattern. Generally, the results suggest that both explicit and implicit attitudes independently contribute to shaping behavior in contexts providing a limited number of choice options, while explicit and implicit attitudes interact in more complex and demanding contexts. These findings underline that the environment in which food choice takes place is an important moderator that could be included as a boundary condition in existing dual process models (c.f. Strack and Deutsch, 2004; Hofmann et al., 2008) and further explored in future (intervention) studies.

## Author Contributions

LK, HG, BR, and HS designed the study. LK analyzed the data. LK and BR drafted the manuscript with critical comments from HG and HS. All authors read and approved the final manuscript.

## Conflict of Interest Statement

The authors declare that the research was conducted in the absence of any commercial or financial relationships that could be construed as a potential conflict of interest.

## Footnotes

1 http://fortune.com/2015/02/25/warren-buffett-diet-coke/

2 www.neurobs.com

3 Positive: 1710, 2070, 5470, 5480, 7502, and 8531; negative: 1525, 2095, 9220, 9600, 9911, and 9925.

4 The pattern of results did not change when all four control variables (hunger, gender, age, and BMI) were included in the analysis.

5 In an additional control analysis, perceived healthiness was included. While a significant positive correlation between perceived healthiness and explicit attitudes was found (*r* = 0.24, *p* = 0.012), no significant relationship between perceived healthiness and food choice emerged in either the binary or the multiple option choice task. Hence, the pattern of results does not change when perceived healthiness is included in the model.
