# Supplementary material for: The Environment Makes a Difference: The Impact of Explicit and Implicit Attitudes as Precursors in Different Food Choice Tasks
Source: Front Psychol. 2016 Aug 29;7:1301. doi: 10.3389/fpsyg.2016.01301 (PMC5002409; doi:10.3389/fpsyg.2016.01301)
Supplement: Supplementary file 1 [file Table_1.DOCX]

Supplementary Material

The Environment Makes a Difference: The Impact of Explicit and Implicit Attitudes as Precursors in Different Food Choice Contexts

Laura M. König*, Helge Giese, Harald T. Schupp, Britta Renner

*** Correspondence:** Laura M. König: laura.koenig@uni-konstanz.de

**Supplementary Table 1**

Results of the Pretest Study: Descriptive Statistics of the Selection Criteria for all Food Pictures (*N* = 22).

|  | % correctly identified | identifiable | | realistic | |
| --- | --- | --- | --- | --- | --- |
|  |  | *M* | *SD* | *M* | *SD* |
| **Confectioneries** |  |  |  |  |  |
| *Chocolate cake | 100.00 | 3.80 | .41 | 3.45 | .69 |
| *Chocolate candies | 100.00 | 3.85 | .37 | 3.75 | .44 |
| Muffin | 190.00 | 3.10 | .79 | 2.75 | .72 |
| *Strawberry cream cake | 100.00 | 3.78 | .43 | 2.50 | 1.15 |
| Tartlet | 183.33 | 3.28 | .67 | 2.78 | .73 |
| **Fruits** |  |  |  |  |  |
| *Apple | 100.00 | 3.84 | .38 | 3.21 | 1.03 |
| *Banana | 100.00 | 3.89 | .32 | 3.39 | .78 |
| Blackberries | 194.74 | 3.68 | .58 | 3.32 | .75 |
| *Orange | 100.00 | 3.84 | .36 | 3.37 | 1.01 |
| Strawberries | 100.00 | 3.83 | .38 | 2.72 | 1.02 |
| **Animal products (main study: meat)** |  |  |  |  |  |
| Fish sticks | 194.74 | 2.63 | .90 | 2.58 | .84 |
| Fried egg | 100.00 | 3.65 | .59 | 2.25 | .91 |
| *Meatballs | 100.00 | 3.44 | .61 | 3.11 | .68 |
| *Sausage | 100.00 | 3.11 | .73 | 2.26 | .73 |
| Schnitzel | 137.50 | 2.44 | 1.15 | 2.50 | .86 |
| *Slice of meat | 100.00 | 2.83 | 1.04 | 2.22 | 1.00 |
| **Side dishes** |  |  |  |  |  |
| Baguette | 100.00 | 3.50 | .61 | 33.25 | .63 |
| *Brown bread | 100.00 | 3.44 | .62 | 3.44 | .78 |
| *Pasta | 100.00 | 3.74 | .45 | 3.47 | .61 |
| *Potatoes | 100.00 | 3.00 | .77 | 2.89 | .90 |
| Rice | 194.74 | 2.79 | .79 | 2.79 | .86 |
| Whole grain bread | 100.00 | 3.28 | .67 | 3.39 | .61 |
| **Vegetables** |  |  |  |  |  |
| *Beans | 100.00 | 3.63 | .50 | 3.32 | .58 |
| *Carrots | 100.00 | 3.56 | .51 | 2.67 | .97 |
| Cucumbers | 183.88 | 3.39 | .70 | 2.56 | .86 |
| Lettuce | 189.47 | 2.79 | .98 | 2.16 | 1.17 |
| *Peas | 100.00 | 3.60 | .60 | 3.20 | .77 |
| Tomatoes | 100.00 | 2.89 | .90 | 1.83 | .92 |

*Note.* * Food item was chosen for main study.
