# Supplementary material for: The Environment Makes a Difference: The Impact of Explicit and Implicit Attitudes as Precursors in Different Food Choice Tasks
Source: Front Psychol. 2016 Aug 29;7:1301. doi: 10.3389/fpsyg.2016.01301 (PMC5002409; doi:10.3389/fpsyg.2016.01301)
Supplement: Supplementary file 2 [file Data_Sheet_1.DOCX]

Supplementary Material

The Environment Makes a Difference: The Impact of Explicit and Implicit Attitudes as Precursors in Different Food Choice Contexts

Laura M. König*, Helge Giese, Harald T. Schupp, Britta Renner

*** Correspondence:** Laura M. König: laura.koenig@uni-konstanz.de

*Supplementary Figure 1. Examples for meals self-served in the multiple option choice task.*
